# Psychosocial Syndemic of suicidal ideation: a cross-sectional study among sexually transmitted infection patients in Shanghai, China

**DOI:** 10.1186/s12889-020-09404-y

**Published:** 2020-08-31

**Authors:** Suping Wang, Yang Ni, Ruijie Gong, Yuan Shi, Yong Cai, Jin Ma

**Affiliations:** 1 School of Public Health, Shanghai Jiao Tong University School of Medicine, No. 227 South Chongqing Road, Shanghai, 200025 China; 2 Shanghai Skin Disease Hospital, Tongji University School of Medicine, No. 1278 Baode Road, Shanghai, 200443 China; 3 Shanghai Xuhui Center for Disease Control and Prevention, No. 50 Yongchuan Road, Shanghai, 200237 China

**Keywords:** Syndemic, Psychosocial problems, Suicidal ideation, Sexually transmitted infection patients

## Abstract

**Background:**

Patients with sexually transmitted infections (STIs) experience difficulties with stability and trust in long-term relationships and have poor mental health, factors that may lead to suicidal ideation. We sought to verify whether psychosocial health problems among patients with STIs were associated with these patients’ suicidal ideation and to examine the syndemic effect of multiple psychosocial problems on suicidal ideation.

**Methods:**

This was a cross-sectional study of 519 STI patients at the Shanghai Skin Disease Hospital. Demographic, psychosocial, and suicidal ideation information about the participants was collected by questionnaire. Logistic regressions were performed to detect the association between demographic variables and suicidal ideation, as well as each individual psychosocial variable and suicidal ideation, and to verify the syndemic effect of psychosocial factors.

**Results:**

Of the participants, 25.0% (130/519) reported having suicidal ideation. In univariable analysis, low self-esteem, loneliness, depression, entrapment, defeat, and unsatisfied interpersonal needs were associated with suicidal ideation. Multivariable analysis found depression (odds ratio [OR]: 4.1; 95% confidence interval [CI]: 2.3–7.2) and entrapment (OR: 2.1; 95%CI: 1.1–4.1) each had a more significant relation with suicidal ideation than the other psychosocial problems examined. STI patients who experienced two or more psychosocial health problems had approximately fourfold odds of suicide ideation (adjusted OR [AOR]: 4.2; 95%CI: 2.6–6.8) compared with those in the non-syndemic group, especially in the high-level (five or more psychosocial problems) group (AOR: 7.0; 95%CI: 3.9–12.5).

**Conclusions:**

The study found the participants had a high rate of suicidal ideation and suffered from severe psychosocial problems. These results show a syndemic effect of psychosocial problems on increasing the odds of suicidal ideation. Our findings suggest an urgent need for efforts to prevent suicidal ideation among STI patients toward improving the social and health conditions of this population.

## Background

According to the World Health Organization, one person in the world commits suicide every 40 s. However, suicide is preventable. Suicidal ideation, defined as thinking about, considering, or planning for suicide [1], may be a prodrome for ultimately committing suicide. Those with suicidal ideation have higher risk for suicide than those without. Suicidal ideation may also indicate a person suffers from great distress and psychological burden. Sexually transmitted infection (STI) patients experience difficulties with stability and trust in long-term relationships [2] and have poor mental health [3–7], factors that may lead to suicidal ideation.

One study in Canada found that 6.7% of STI patients had suicidal ideation and mental health needs over the previous 12 months [8]. Other investigations have examined people living with the human immunodeficiency virus/acquired immunodeficiency syndrome (HIV/AIDS; PLWHA), the range of suicidal ideation was 27.2 to 43.1% [9–12].

In one survey of STI patients in Jamaica, 65.5% of participants screened positive for at least one psychosocial problem, including depression and suicidal ideation [8]. Previous studies also revealed the association between certain psychosocial problems and suicidal ideation. Additionally, studies have confirmed that low self-esteem and depression are the principal factors behind suicidal ideation [13–23], and more-current articles have reported that loneliness, unsatisfied interpersonal needs, entrapment, defeat, and poor social support are also strong predictors [17, 19, 24–26]. One study in China reported poor social support as the strongest predictor of suicidal ideation, depression and low self-esteem were also strong predictors [24]. In two recent studies, one in the United States reported that loneliness and poor social support might represent the most important components of connectedness because they were found to be associated with depression severity and suicidality; the other study in Swaziland reported feeling lonely as a risk factor for suicidal ideation [27, 28]. Defeat and entrapment are key variables of Williams’ cry of pain model of suicide and central to O’Connor’s integrated motivational–volitional (IMV) model [29, 30]. Recently, Joiner proposed the interpersonal theory of suicide (IPTS) [31], which asserts that suicidal ideation emerges when individuals experience thwarted belongingness (loneliness and lack of reciprocal care) and perceived burdensomeness (perceived liability to others and self-hate).

Notably, individuals with several psychosocial problems may have magnified suicidal ideation [9, 32, 33]. We thus introduce the syndemic theory to this discussion. “Syndemic” as a term was first proposed by Singer to describe “synergistically related” epidemics that cluster and arise from harmful social conditions [34]. A syndemic effect, or synergistic epidemic, is the aggregation of two or more concurrent or sequential epidemics or disease clusters in a population. This effect exacerbates the disease prognosis and burden. To our knowledge, most research on the mechanism of suicidal ideation among STI patients (including PLWHA) has focused on a single psychosocial factor [12, 35]. These studies also address behavioral habits, risky sexual behaviors, unpleasant sexual experiences, and psychosocial problems. There is inherent difficulty in obtaining truthful answers because such matters involve a high degree of privacy. In China’s mainstream culture this issue is a particular challenge. Some research has even considered STIs as a factor behind the syndemic effect on suicidal ideation, whereas the present study focused particularly on STI patients’ psychosocial problems [33, 34, 36]. Only one study has discussed the association between a syndemic effect of psychosocial complements (depression, self-esteem, and social support) and suicidal ideation among HIV+ patients in Nanjing, China [33]. Among other populations in China, our team identified a psychosocial syndemic effect (self-esteem, depression, social support, and loneliness) and suicidal ideation in men who have sex with men (MSM) [12]. The present study used defeat, entrapment, and poor interpersonal need as psychosocial factors, in addition to depression, low self-esteem, loneliness, and poor social support, to determine whether the syndemic effect would be similar among STI patients while considering the association with suicidal ideation.

In this study, we aimed to verify three hypotheses: [1] STI patients suffer several psychosocial problems and a high rate of suicidal ideation [2]; suicidal ideation is strongly associated with psychosocial problems; and [3] there is a psychosocial syndemic effect in suicidal ideation among STI patients.

## Methods

### Participants

This cross-sectional study, started in November 2017, was conducted for 6 months among STI clinic patients in two branch institutes of the Shanghai Skin Disease Hospital: Qiujiang Road and Baode Road. This hospital specializes in STIs and is one of the premier medical institutes in this field.

In accordance with the Law of the People’s Republic of China on the Prevention and Treatment of Infectious Diseases, “STI patients” herein refers to those with any of five types of STIs that need preventive measures: syphilis, gonorrhea, genital warts, genital herpes, and HIV. All diagnoses (including clinical diagnoses and laboratory diagnosis) were conducted by sexually transmitted disease (STD) doctors at the hospital. Individuals who met the following criteria were invited to take part: ≥18 years old, clinically diagnosed with an STI disease, able to read the informed consent form, and no participation in a similar study in the previous 6 months.

Patients who had any of the following criteria, were excluded: severe mental or cognitive impairment (e.g., neurosyphilis), unconsciousness, or disinclination to participate (*n* = 88).

Assuming 30.0% prevalence of lifetime suicidal ideation in STI patients, using alpha of 0.05 and a relative error for sampling of 0.15, we calculated a required sample size of 415 [7, 33]. To allow for a 30% non-response rate, a total of 540 patients were recruited to participate, and 519 (96.1%) valid questionnaires were collected.

### Ethics

The Shanghai Jiao Tong University School of Medicine Public Health and Nursing Ethics Committee approved the study (approval number: SJUPN-201702). Background information on the survey was given orally to all participants, after which they were given written informed consent forms that set out the study’s goal, procedures, and potential risks. Participants signed informed consent forms before the study began. During the recruitment, participants were free to ask any questions and to withdraw.

### Procedure

Our survey team signed cooperation agreements with the Shanghai Skin Disease Hospital before the investigation. All the doctors worked in the STD Department (inpatient or outpatient) were recruited and informed about the survey beforehand. All the investigators were students (seniors or postgraduates) from Shanghai Jiao Tong University School of Medicine. They received pre-survey training and several in-person reviews throughout the study. The training also incorporated quality-control strategies, such as reexamining and investigating the questionnaires and resolving issues that may arise during the fieldwork.

The doctors informed each participant who met the inclusion criteria about the survey. After initial approval, participants were asked to enter a separate room to attend an interview conducted by our investigators. The interviews were divided into two parts: [1] Our investigators informed participants about the study goal, study procedure, and potential risks before participants signed the informed consent form [2]. The participants completed anonymous questionnaires in a separate room to protect their privacy and to confirm the validity of the questionnaire data. The investigators only provided assistance upon request. Each participant received 80 RMB (approximately USD 12) cash for his/her participation.

Our investigators went to the two branch institutes of the Hospital every Wednesday and Saturday during the study period. Patients who visited one of the two branch institutes to see a doctor for STI treatment on a Wednesday and Saturday (when our investigators were present) were regarded as potential objects. Doctors at the institutes assessed whether patients met our eligibility criteria and inquired about participation. No appointment system was implemented at the two institutes, so our investigators meeting the patients was accidental. Thus, we employed accidental sampling.

### Measures

#### Demographic variables

Demographic variables included age, sex, educational level, marital status, residency, income, insurance, sexual orientation, and HIV status.

#### Suicidal ideation

Suicidal ideation was measured via one question [12]: “Have you ever thought about committing suicide?” (hereinafter defined as suicidal ideation; 0 = no, 1 = yes).

### Psychosocial variables

#### Self-esteem

Self-esteem was assessed based on the 10-item Rosenberg Self-Esteem Scale (SES) [37]. Negative statements such as “all in all, I am inclined to feel that I am a failure” required a reverse in score (e.g., 0 = 3, 1 = 2, 2 = 1, 3 = 0) before adding to the total. The Chinese version was used and low self-esteem was indicated with a result < 29 (the norm for a Chinese population in China was 28.75 [38]) (Cronbach’s α: 0.847; range: 12–40).

#### Loneliness

The initial version of the UCLA Loneliness Scale used 20 items (e.g., “I lack companionship”) designed to estimate participants’ loneliness and related emotional states [39]. Hays and DiMatteo identified a highly correlated alternative of eight items (eight-item UCLA Loneliness Scale: ULS-8), to achieve similar reliability but reduce the respondent’s time burden and improve data quality [40]. In this study, we used the Chinese version of ULS-8 [41] (Cronbach’s α: 0.820; range: 8–32). The more loneliness the individual felt, the higher that person would score, with a cutoff point set at the 75th percentile; a score of 18.

#### Depression

Depression level was gauged using the Chinese version of Patient Health Questionnaire-9 (PHQ-9), a brief screening tool comprising nine items that match diagnosis criteria in the *Diagnostic and Statistical Manual of Mental Disorders, Fourth Edition*. Individuals were asked to recall how often they had experienced troubling problems (e.g., “little interest or pleasure in doing things”) over the preceding 2 weeks, scoring from 1 (not at all) to 4 (nearly every day) [42]. A cutoff point of an algorithm score of 5 showed good screening performance within various settings [43] (Cronbach’s α: 0.910; range: 0–27).

#### Entrapment

Evaluation of entrapment was quantified using the Chinese version of 16-item Entrapment Scale (ES) [44, 45], which reflected the escape motivation triggered either by perception of the outside world (e.g., “I am in a relationship I can’t get out of”) or internal feelings (e.g., “I want to get away from myself”) (Cronbach’s α: 0.965; range: 0–64). Higher than the 75th percentile—a score of 21—was defined as high entrapment.

#### Defeat

For the 16-item Chinese version of Defeat Scale (DS) [44, 46], participants were asked about how they had thought about themselves in the preceding 7 days. Three items [2, 4, 9] were recoded before computing. Their scores were calculated by summing the items for each scale (scored 0–4) (Cronbach’s α: 0.886; range: 0–56). Higher than the 75th percentile—a score of 23—was defined as high defeat.

#### Interpersonal needs

Interpersonal needs were measured using the 15-item Chinese version of the Interpersonal Needs Questionnaire (INQ-15), which measures perceived burdensomeness (items 1 to 6) and thwarted belongingness (items 7 to 15) [47, 48]. Respondents rated how often they felt a certain way (e.g., “These days, the people in my life would be better off if I were gone”), scored from 1 (not at all true for me) to 7 (very true for me). Six items [7, 8, 10, 13, 49] were recoded before computing. Higher than the 75th percentile score of 49 was defined as interpersonal needs being unsatisfied (Cronbach’s α: 0.855; range: 15–96).

#### Perceived social support

The Chinese version of the Multidimensional Scale of Perceived Social Support (MSPSS) is a 12-item, seven-point Likert scale based on the self-reported measure of support received from family, friends, and/or other people of special significance (e.g., “There is a special person with whom I can share joys and sorrows”) [50]. Scoring ranged from 1 (very strongly disagree) to 7 (very strongly agree). A higher score indicated better social support, and the 25th percentile—a score of 59—was adopted as the cutoff point (Cronbach’s α: 0.947; range: 12–84).

#### Psychosocial problems

Psychosocial problems included low self-esteem, high loneliness, high depression, high entrapment, high defeat, unsatisfied interpersonal needs, and low perceived social support. Low self-esteem was defined as individuals who scored ≤29 points on the SES. High loneliness was defined as those who scored > 18 on the ULS-8. High depression was defined as individuals who scored > 5 points on the PHQ-9. High entrapment was defined as those who scored > 21 points on the ES. High defeat was defined as individuals who scored > 23 points on the DS. Unsatisfied interpersonal need was defined any those who scored > 49 points on the INQ-15. Low perceived social support was defined as individuals who scored ≤59 points on the MSPSS.

#### Syndemic effect of psychosocial variables

Two or more concurrent psychosocial problems in a participant indicated a syndemic phenomenon [51]. The syndemic effect was established via the number of concurrent problems. Five or more indicated a high level of syndemic effect; otherwise, the participant had a low level.

### Statistical analysis

Statistical analysis was performed using IBM SPSS Statistics for Windows, Version 22.0 (IBM Corp., Armonk, NY, USA). First, baseline descriptive statistics were calculated to summarize demographic characteristics, suicidal ideation, and psychosocial variables. Univariable analysis was then conducted via binary logistic regression to detect the association between demographic variables and suicidal ideation, and between univariable psychosocial variable and suicidal ideation. After adjusting for all significant demographic variables, univariate logistic regression analysis was performed to examine the psychosocial syndemic effect on suicidal ideation among these STI patients. Multivariable logistic regression was then used to evaluate all seven psychosocial problems associated with suicidal ideation, after adjusting for significant demographic variables. In the final stage, syndemic count variables were created by counting each individual’s number of psychosocial health problems, and different groups were established based on the number of variables.

## Results

### Demographic characteristics of participants

In all, 519 patients (median age, 34.0 years) were investigated: 25.0% (130/519) had experienced suicidal ideation; 9.6% (50/519) were HIV-positive; 47.6% (247/519) were diagnosed with genital warts; and 23.9% (124/519) were diagnosed with syphilis (Table 1).

**Table 1:** Demographic characteristics of the participants and associations with suicidal ideation(*N* = 519)

Demographic Characteristics	Number of participants		Had suicidal ideation		
	n/N	column(%)	n/N	row(%)	OR(95%CI)
**Case**					
Outpatient	401/519	77.3	98/401	24.4	0.9 (0.5–1.4)
Inpatient	118/519	22.7	32/118	27.1	1
**Age group**^**a**^					
< 25	54/519	10.4	25/54	46.3	4.7 (1.6–13.9)**
25–40	326/519	62.8	85/326	26.1	1.9 (0.7–5.2)
41–59	107/519	20.6	15/107	14.0	0.9 (0.3–2.6)
≥ 60	32/519	6.2	5/32	15.6	1
**Sex**					
Male	232/519	44.7	50/232	21.6	0.7 (0.5–1.1)
Female	287/519	55.3	80/287	27.9	1
**Education**^**b**^					
Middle School or less	114/519	22.0	38/114	33.3	1.6 (0.9–2.5)
High School	98/519	18.9	18/98	18.4	0.7 (0.4–1.3)
College degree or above	307/519	59.1	74/307	24.1	1
**Current Marital Status**					
Married	314/519	60.5	63/314	20.1	0.5 (0.3–0.8)**
Divorce	32/519	6.2	8/32	25.0	0.7 (0.3–1.6)
Widowed	10/519	1.9	5/10	50.0	2.0 (0.6–7.3)
Unmarried (Single)	163/519	31.4	54/163	33.1	1
**Income (RMB)**^**c**^					
≥ 12,001	123/519	23.7	22/123	17.9	0.4 (0.2–0.8)**
6001–12,000	168/519	32.4	41/168	24.4	0.6 (0.3–1.1)
3001–6000	155/519	29.9	42/155	27.1	0.7 (0.4–1.3)
≤ 3000	73/519	14.0	25/73	34.2	1
**Residence Status**^**d**^					
Local	227/519	43.7	41/227	18.1	0.6 (0.4–1.1)
Stay less than 1 year	85/519	16.4	29/85	34.1	1.5 (0.8–2.7)
Stay 1–5 year	65/519	12.5	24/65	36.9	1.7 (0.9–3.2)
Stay more than 5 year	142/519	27.4	36/142	25.4	1
**Self-reported sexual orientation**					
Not sure	19/519	3.7	6/19	31.6	1.5 (0.6–4.2)
Homosexual	30/519	5.8	15/30	50.0	3.4 (1.6–7.1)**
Bisexual	13/519	2.5	4/13	30.8	1.5 (0.5–5.0)
Heterosexual	457/519	88.0	105/457	23.0	1
**Insurance**					
Have	466/519	89.8	111/466	23.8	0.6 (0.3–1.0)
No	53/519	10.2	19/53	35.8	1
**HIV Status**^**e**^					
Positive	50/519	50/519	18/50	36.0	1.7 (0.9–3.2)
Unknown	167/519	167/519	43/167	25.7	0.8 (0.5–1.3)
Negative	302/519	302/519	69/302	22.8	1
**Type of STIs**					
Gonorrhea	14/519	2.7	2/14	14.3	0.3 (0.1–1.5)
Syphilis	124/519	23.9	29/124	23.4	0.5 (0.3–1.1)
Genital warts	247/519	47.6	61/247	24.7	0.6 (0.3–1.1)
Genital herpes	12/519	2.3	3/12	25.0	0.6 (0.1–2.5)
Others	72/519	13.9	17/72	23.6	0.5 (0.2–1.2)
HIV	50/519	9.6	18/50	36.0	1
**Have had suicidal ideation in life time**					
Yes	130/519	25.0			
No	389/519	75.0			

^a,b,c,d^Classification in accordance with China’s regulations

^e^HIV status: unknown including not detected

**p < 0.01

### Psychosocial health conditions

Of the participants, 24.9% (129/519) were classified as lonely, 23.7% (123/519) at a high level of entrapment, 24.1% (125/519) with defeat, and 22.9% (119/519) as having unmet interpersonal needs. Of the participants, 25.8% (134/519) lacked social support. About half of the participants (48.4%; 251/519) showed depression via their PHQ-9 scores. A total of 38.9% (202/519) had a low level of self-esteem (Table 2).

**Table 2:** Psychosocial health conditions among participants (N = 519)

Psychosocial Health Conditions	n/N	column(%)	Median	Media	SD
**Self-esteem**^a^					
High level (score ≥ 29)	317/519	61.1	29.0	29.7	4.2
Low level (score < 29)	202/519	38.9			
**Loneliness**^b^					
High level (score > P_75_,18)	129/519	24.9	14.0	14.8	5.0
Low level (score ≤ P_75_,18)	390/519	75.1			
**Depression**^c^					
Yes (score > 5)	251/519	48.4	5.0	6.3	5.4
No (score ≤ 5)	268/519	51.6			
**Entrapment**^b^					
High level (score > P_75_,21)	123/519	23.7	8.0	12.3	13.2
Low level (score ≤ P_75_,21)	396/519	76.3			
**Defeat**^b^					
High level (score > P_75_,23)	125/519	24.1	14.0	16.5	11.0
Low level (score ≤ P_75_,23)	394/519	75.9			
**Interpersonal need**^b^					
High level (score > P_75_,49)	119/519	22.9	34.0	36.2	14.7
Low level (score ≤ P_75_,49)	400/519	77.1			
**Perceived social support**^d^					
High level (score > P_25_,59)	385/519	74.2	70.0	66.6	14.0
Low level (score ≤ P_25_,59)	134/519	25.8			

^a^cutoff of SES: 29

^b^cutoff: P_75_

^c^cutoff: 5

^d^cutoff: P_25_

### Demographic characteristics associated with suicidal ideation

Table 1 shows the univariable association between participants’ demographic characteristics and their suicidal ideation. Four demographic variables (age, marital status, income, and self-reported sexual orientation) showed significant relation to suicidal ideation. Participants aged < 25 years had more than four times (odds ratio [OR]: 4.7; 95% confidence interval [CI]: 1.6–13.9) higher reports of suicidal ideation than those aged ≥60 years. Married participants, compared with unmarried, were less likely to have suicidal ideation (OR: 0.5; 95%CI: 0.3–0.8). The highest income group was less likely than the lowest to have suicidal ideation (OR: 0.4; 95%CI: 0.2–0.8). Homosexual respondents were more likely to report suicidal ideation than heterosexual respondents (OR: 3.4; 95%CI: 1.6–7.1).

### Psychosocial problems associated with suicidal ideation

Table 3 summarizes the binary regression results. After adjusting for age, marital status, income, and self-reported sexual orientation, six of the seven total psychosocial variables constructed among syndemic psychosocial factors showed statistical significance with experiencing suicidal ideation. Participants demonstrating a higher level of loneliness (adjusted odds ratio [AOR]: 2.4; 95%CI: 1.5–3.7), depression (AOR: 6.1; 95%CI: 3.7–10.1), entrapment (AOR: 4.5; 95%CI: 2.9–7.2), defeat (AOR: 3.5; 95%CI: 2.2–5.3), unsatisfied interpersonal needs (AOR: 1.9; 95%CI: 1.2–3.1), or a low level of self-esteem (AOR: 2.1; 95%CI: 1.4–3.3) were at increased risk for suicidality. Social support was not significantly associated with suicidal ideation among these STI patients. However, the multivariable logistic regression showed that only two psychosocial factors remained significant: entrapment (ORs obtained from forward stepwise multivariable logistic regression [ORm]: 2.1; 95% CI: 1.1–4.1) and depression (ORm: 4.1; 95% CI: 2.3–7.2).

**Table 3:** Psychosocial problems associated with suicidal ideation among participants (N = 519)

Psychosocial Problems	n/N	row(%)	OR^**a**^ (95%CI)	AOR^**b**^ (95%CI)	ORm^**c**^ (95%CI)
**Self-esteem**					
Low level (score < 29)	71/202	35.1	2.4 (1.6–3.6)**	2.1 (1.4–3.3)**	
High level (score ≥ 29)	59/317	18.6	1	1	
**Loneliness**					
High level (score > P_75_,18)	52/129	40.3	2.7 (1.8–4.2)**	2.4 (1.5–3.7)**	
Low level (score ≤ P_75_,18)	78/390	20.0	1	1	
**Depression**					
Yes (score > 5)	104/251	41.4	6.6 (4.1–10.6)**	6.1 (3.7–10.1)**	4.1 (2.3–7.2)**
No (score ≤ 5)	26/268	9.7	1	1	
**Entrapment**					
High level (score > P_75_,21)	63/123	51.2	5.2 (3.3–8.0)**	4.5 (2.9–7.2)**	2.1 (1.1–4.1)*
Low level (score ≤ P_75_,21)	67/396	16.9	1	1	
**Defeat**					
High level (score > P_75_,23)	56/125	44.8	3.5 (2.3–5.4)**	3.5 (2.2–5.3)**	
Low level (score ≤ P_75_,23)	74/394	18.8	1	1	
**Interpersonal need**					
High level (score > P_75_,49)	42/119	35.3	1.9 (1.2–3.0)**	1.9 (1.2–3.1)**	
Low level (score ≤ P_75_,49)	88/400	22.0	1	1	
**Perceived social support**					
Low level (score ≤ P_25_,59)	39/385	10.1	1.3 (0.9–2.1)	1.3 (0.8–2.1)	
High level (score > P_25_,59)	91/134	67.9	1	1	

^a^Odds ratios without adjusted any confounder

^b^Odds ratios adjusted for significant demographic variables including age, marital status, income, self-reported sexual orientation

^c^Odds ratios obtained from forward stepwise multivariable logistic regression using significant variables of the univariate analysis as input

*p < 0.05

**p < 0.01

### Verification of Syndemic effect of psychosocial variables

Table 4 shows the results of the final syndemic analysis. Generally, it was found that having at least two concurrent psychosocial health problems had a syndemic effect in fusing suicidal ideation (AOR: 4.9; 95%CI: 3.1–7.8). The low-level group (AOR: 4.2; 95%CI: 2.6–6.8) and high-level group (AOR: 7.0; 95%CI: 3.9–12.5) showed a prominent syndemic effect compared with those in the non-syndemic group.

**Table 4:** Association between the number of psychosocial problems and suicidal ideation among participants (N = 519)

	n/N	Column(%)	had suicidal ideation		
			n/N	row(%)	AOR (95%CI)
**Model 1**					
**Have a syndemic**					
Yes	257/519	49.5	100/257	38.9	4.9 (3.1–7.8)**
No	262/519	50.5	30/262	11.5	1
**Model 2**					
**Number of psychosocial problems**					
5 or more psychosocial problems	80/519	15.4	38/80	47.5	7.0 (3.9–12.5)**
2 to 4 psychosocial problems	177/519	34.1	62/177	35.0	4.2 (2.6–6.8)**
No	262/519	50.5	30/262	11.5	1

## Discussion

The rate of lifetime suicidal ideation among STI patients in Shanghai in our study was 25.0% (130/519). Among people living with HIV/AIDS in the present study, 18 showed suicidal ideation (36.0%; 18/50). In this study, suicidal ideation was independently associated with six psychosocial problems, though not social support. Our most important finding is the syndemic effect of entrapment and defeat in addition to depression, self-esteem, interpersonal needs, loneliness, and social support in suicidal ideation among STI patients, especially in those who had five or more concurrent psychosocial problems.

The difference in suicidal ideation prevalence between our sample (25.0%) and that of a Canadian sample (6.7%) [8] could be explained as follows. First, we measured lifetime suicidal ideation and the Canadian research team measured suicidal ideation during the previous 12 months. In addition, the patient demographics in the two studies were substantially different. For example, 65% of the patients in the Canadian study were in a sexual minority, whereas 88% of the patients in our study were heterosexual. The suicidal ideation prevalence of PLWHA in our study was 36.0%. This is similar to previously reported prevalences, which range from 27.2 to 43.1% [9–12]. The significant demographic variables associated with suicidal ideation agreed with findings in other studies [52]. Young participants were at a higher risk of suicidal ideation, possibly owing to higher impulsivity, lower ability to assume responsibility, and lower psychological ability to counteract feelings of vulnerability when faced with harsher social discrimination or mistreatment, which agreed with findings in a study in Swaziland and another in China [27, 53]. Instrumental and emotional support, and financial ability to enable better treatment resources, may explain marital status and higher income as protective factors according to some studies of STI patients in China [54–56]. Research has shown higher suicidality prevalence among male homosexual orientation, in line with the results of our present research [57, 58]. HIV status was not associated with suicidal ideation, which alerts us that STI patients without HIV may suffer the same high rate of ideation as in HIV patients [59]. Consistent with previous studies, our study found suicidal ideation was independently associated with low self-esteem, depression, loneliness, entrapment, defeat, and interpersonal need. According to cognitive theory, low self-esteem is processed in a typically negative manner, which leads to negative self-appraisal and later to suicidal ideation [60]. As suicidal ideation is among the diagnostic criteria for depression, its presence in and of itself will necessarily increase the number of depressive symptoms. Feeling lonely was associated with distress, which is strongly associated with generalized anxiety, panic attacks, and suicidal ideation [61]. The association between loneliness and suicidality supports the theory that thwarted belongingness and perceived burdensomeness are major determinants of suicidality [31]. The relationship between defeat, entrapment, and suicidal ideation is the motivational phase of the IMV model [30], and also established key variables within Williams’ cry of pain theory of suicide [29]. Previous research is equivocal regarding the relationship between social support and suicidal ideation.

When we included all psychosocial variables in a single model, the association with suicidal ideation was removed for most variables, though it remained strongest for depression and entrapment. This should not be surprising, considering suicidal ideation and behavior are known outcomes of feeling trapped in a stressful situation, with no evident escape or rescue [62]; this result has been found in diverse populations and in the context of various disorders and research methodologies [56, 57]. Despite prevalence of poor psychosocial status among STI patients, huge gaps are still visible in concern and service, due to inadequate information and emotional support, and a shortage of qualified professional psychosocial treatment [13, 63–65].

To show that suicidal ideation also co-occurs among STI patients, we have extended previous research that confirmed there was a syndemic effect on suicidal ideation in MSM [32]. We confirmed all seven psychosocial health problems tend to co-occur and act to raise risk levels for suicidal ideation in these patients. However, the measurement of the syndemic construct is not invariant across groups; therefore, the construct’s meaning also differs. A previous study showed syndemics are in fact a general human phenomenon, but their composition differs, and the consequences are felt most deeply by those in the minority, such as MSM and men who have sex with men and women [36]. These results support the notion that syndemic theory has the potential to advance research, theory, and interventions related to suicidal ideation in this population.

Prevention and intervention efforts should be designed and implemented to reduce suicidal ideation in STI patients. Especially in hospitals, psychological counseling and interventions are needed to improve individuals’ mental well-being and reduce suicidal ideation [66–69]. In China, such efforts, especially well-designed psychosocial programs, are still strongly needed for most STI patients and PLWHA [70–73].

### Limitations and future research

Several limitations should be considered in interpreting the present results. First, cross-sectional surveys have difficulty determining causality; therefore, a prospective study assessing recent STI diagnoses and suicidal ideation would be beneficial. Second, although the participants came from a representative hospital, the sample size was not especially large; multi-center research is needed. Third, although privacy was ensured, investigators were trained, and doctors’ cooperation ensured survey quality, self-reported and recall bias were unavoidable. Fourth, our findings do not indicate the incidence of suicidal ideation in newly diagnosed and revisited patients; future studies are needed to focus on and explore the differences between the two. Fifth, there was selection bias. For example, we excluded from this study neurosyphilis patients with severe mental or cognitive impairment; patients who had experienced associated stigma may also have been more reluctant to participate. And accidental sampling reduces the possibility of replicability and generalization. Sixth, we used a self-reported binary scale assess lifetime suicidal ideation, which potentially underestimated its prevalence. Finally, the syndemic effect examined classification variables using a cutoff point, which may not supply an adequate amount of information, although the results for continuous variables were similar.

## Conclusions

This study adds to the literature in several important ways. We demonstrated that not only PLWHA but also other STI patients have high rates of suicidal ideation, and this population suffers from severe psychosocial problems. This study identified a syndemic effect of psychosocial problems on increasing odds of suicidal ideation. The collective findings suggest that greater attention should be paid to STI patients’ psychosocial well-being; especially in hospitals. Efforts to prevent suicidal ideation, as well as other mental problems, among STI patients are therefore urgently needed to improve the social and health conditions for this population.

## Data Availability

The datasets used and/or analysed during the current study are available from the corresponding author on reasonable request.

## Abbreviations

STD: Sexually transmitted disease
STI: Sexually transmitted infection
HIV: Human immunodeficiency virus
AIDS: Acquired Immune deficiency syndrome
PLWHA: People living with HIV/AIDSIMV model: integrated motivational–volitional model
IPTS: Interpersonal theory of suicide
MSM: Men who have sex with men
SES: Rosenberg Self-esteem Scale
ULS-8: Eight-item UCLA Loneliness Scale
PHQ-9: Nine-item Patient Health Questionnaire
ES: 16-item Entrapment Scale
DS: 16-item Defeat Scale
INQ-15: 15-item Interpersonal Need Questionnaire
MSPSS: Multidimensional Scale of Perceived Social Support
OR: Odd ratio
CI: Confidence interval
AOR: Adjusted odd ratio
ORm: Odds ratios obtained from forward stepwise multivariable logistic regression
